# Exome sequencing to explore the possibility of predicting genetic susceptibility to the joint occurrence of polycystic ovary syndrome and Hashimoto’s thyroiditis

**DOI:** 10.3389/fimmu.2023.1193293

**Published:** 2023-07-20

**Authors:** Natalia Zeber-Lubecka, Katarzyna Suchta, Maria Kulecka, Anna Kluska, Magdalena Piątkowska, Michal J. Dabrowski, Katarzyna Jankowska, Monika Grymowicz, Roman Smolarczyk, Ewa E. Hennig

**Affiliations:** ^1^ Department of Gastroenterology, Hepatology and Clinical Oncology, Centre of Postgraduate Medical Education, Warsaw, Poland; ^2^ Department of Genetics, Maria Sklodowska-Curie National Research Institute of Oncology, Warsaw, Poland; ^3^ Department of Gynaecological Endocrinology, Medical University of Warsaw, Warsaw, Poland; ^4^ Institute of Computer Science, Polish Academy of Sciences, Warsaw, Poland; ^5^ Department of Endocrinology, Centre of Postgraduate Medical Education, Warsaw, Poland

**Keywords:** polycystic ovary syndrome, autoimmune thyroid disease, Hashimoto thyroiditis (HT), whole-exome sequencing (WES), prediction model, immune response, female meiosis, cilia

## Abstract

A large body of evidence indicates that women with polycystic ovary syndrome (PCOS) have a higher risk of developing Hashimoto’s thyroiditis (HT) than healthy individuals. Given the strong genetic impact on both diseases, common predisposing genetic factors are possibly involved but are not fully understood. Here, we performed whole-exome sequencing (WES) for 250 women with sporadic PCOS, HT, combined PCOS and HT (PCOS+HT), and healthy controls to explore the genetic background of the joint occurrence of PCOS and HT. Based on relevant comparative analyses, multivariate logistic regression prediction modeling, and the most informative feature selection using the Monte Carlo feature selection and interdependency discovery algorithm, 77 variants were selected for further validation by TaqMan genotyping in a group of 533 patients. In the allele frequency test, variants in *RAB6A*, *GBP3*, and *FNDC7* genes were found to significantly (*p_adjusted_* < 0.05) differentiated the PCOS+HT and PCOS groups, variant in *HIF3A* differentiated the PCOS+HT and HT groups, whereas variants in *CDK20* and *CCDC71* differentiated the PCOS+HT and both single disorder groups. TaqMan genotyping data were used to create final prediction models, which differentiated between PCOS+HT and PCOS or HT with a prediction accuracy of AUC = 0.78. Using a 70% cutoff of the prediction score improved the model parameters, increasing the AUC value to 0.87. In summary, we demonstrated the polygenic burden of both PCOS and HT, and many common and intersecting signaling pathways and biological processes whose disorders mutually predispose patients to the development of both diseases.

## 1 Introduction

Both polycystic ovary syndrome (PCOS) and Hashimoto’s thyroiditis (HT) are common endocrinopathies, affecting 5-20% of the female population of reproductive age (1, 2), with a prevalence that varies depending on the ethnicity of the population and the diagnostic criteria used (3, 4). PCOS is a heterogeneous disorder characterized mainly by hyperandrogenism, ovarian dysfunction often manifested as chronic oligo- or anovulation, and polycystic ovarian morphology (PCOM) on pelvic ultrasonography. Because two of these three features must be present for PCOS diagnosis (Rotterdam criteria) (5), their different combinations determine the various phenotypes of the disease (6).

HT is an organ-specific T cell-mediated autoimmune disorder in which an autoimmune attack targeting components of the thyroid gland can lead to a decreased production of thyroid hormones (hypothyroidism) (3). HT is considered the main cause of hypothyroidism, although it can persist for years without noticeable thyroid dysfunction (7). As a result, HT has a clinically heterogeneous presentation, ranging from the presence of thyroid antibodies but normal thyroid function (euthyroidism) to subclinical hypothyroidism, defined as a serum thyroid-stimulating hormone (TSH) level above the reference limit and a normal level of free thyroxine (fT_4_), and finally, overt hypothyroidism, in which the fT_4_ level is reduced below the normal limit and TSH is further increased (8, 9). In most cases, HT eventually progresses to hypothyroidism, even though patients were euthyroid or even transiently hyperthyroid (hashitoxicosis) at the time of presentation (10). However, it should be noted that the definition of HT is not unambiguous and some authors define HT as hypothyroidism and elevated levels of antithyroid antibodies. Nearly all patients with HT (90-95%) have antibodies against thyroid peroxidase (TPO) and 60-80% have antibodies against thyroglobulin (Tg) (11, 12). In general, the diagnostic criteria for HT are based on the detection of elevated serum levels of anti-TPO and/or anti-Tg antibodies and a typical hypoechogenic pattern in the thyroid gland on ultrasound imaging (13).

PCOS and HT share several common symptoms and may be accompanied by similar endocrine and metabolic disorders, including inflammation, insulin resistance, and dyslipidemia, which contribute to the increased risk of obesity, diabetes, cardiovascular disease, and cancer (14–17). Both disorders are the leading causes of reproductive complications and infertility in women of childbearing age (18). A growing number of studies have indicated a higher incidence of various thyroid disorders in patients with PCOS than in the general population (19, 20). According to several meta-analyses, HT occurs approximately three times more often among patients with PCOS than among healthy women, with a slightly higher prevalence in Asian populations than in European and South American populations (21–24). Interestingly, a higher risk of developing PCOS was observed among Asian patients with newly diagnosed HT but was lower than the risk of developing HT among PCOS patients in the same population (25).

While the close interaction between PCOS and HT seems indisputable, its causes are not entirely clear. Current research has shown that epigenetic changes may play a leading role in the etiology of both diseases. In turn, such post-translational modifications of the genome depend on the environmental changes during the lifetime (26–28). Growing evidence suggests that environmental factors can trigger epigenetic modifications that may lead to the development of the disease in genetically susceptible individuals (29–31). Given the strong genetic component in the heritability of PCOS and HT, exceeding 70% (32, 33), it can be assumed that some elements of their genetic backgrounds are common or mutually predispose to the joint occurrence of these diseases. Although many candidate gene and genome-wide association studies (GWAS) have been performed for each of these diseases (19, 34–36), genetic variants predisposing individuals to the joint occurrence of both disorders have not yet been extensively studied. To date, only a few polymorphisms contributing to both PCOS and HT have been proposed, including the most convincingly described polymorphisms in *FBN3*, *CYP1B1*, and *GNRHR*, and GWAS-selected single nucleotide polymorphisms (SNPs) in *FSHR* and *INSR* which are common in PCOS and HT (17, 19, 37).

Despite many efforts, PCOS- or HT-related variants identified by GWAS account for only a small proportion of the estimated disease heritability (38–40), likely due to the specificity of the GWAS technique, which primarily identifies SNPs in non-coding regions as common markers of linkage disequilibrium with causal variants. PCOS and HT are highly polygenic disorders, in which both common and rare variants may account for multifactorial susceptibility. Unlike GWAS, whole-exome sequencing (WES) allows the identification of variants in the coding region, which are often rare and have a functional effect (41). Several new variants have been discovered in studies using WES for both familial and sporadic cases of PCOS and HT (42–49), confirming that this approach can be effective in discovering genes underlying complex diseases. To date, WES has not been employed to search for susceptibility variants related to the risk of joint occurrence of PCOS and HT. In this study, we performed WES to investigate the genetic background and biological pathways associated with both disorders and explored the possibility of predicting the genetic susceptibility to their joint occurrence.

## 2 Materials and methods

### 2.1 Ethics statement

All enrolled patients and controls were Polish Caucasians. The local ethics committee approved the study (Medical University of Warsaw, No: KB/200/2015 and Centre of Postgraduate Medical Education, No: 77/PB/2017, Warsaw, Poland), and all participants provided written informed consent before participating in the study. The study protocol conformed with the ethical guidelines of the 1975 Declaration of Helsinki.

### 2.2 Study population

A total of 571 women aged 15-45 were recruited for this study. Of these, 250 were included in WES: 70 patients with PCOS (P group), 71 with HT (H group), 71 with both PCOS and HT (P+H group), and 38 healthy women in the control group (K group). To ensure the greatest possible homogeneity of the study groups, stricter inclusion and exclusion criteria were used to recruit patients. Patients included in the WES must meet all three Rotterdam criteria (5) for the diagnosis of PCOS: chronic anovulation or infrequent ovulation, hyperandrogenism presenting as elevated androgenic hormone levels, and the presence of PCOM on ultrasound examination. A serum progesterone (PRG) concentration of ≤ 3 ng/ml on the 22^nd^ to 24^th^ day of the cycle confirmed an anovulatory cycle. Oligomenorrhea was defined as a menstrual cycle duration > 35 days, and secondary amenorrhea was defined as a lack of menstrual bleeding for over six months. Clinical hyperandrogenism manifests as hirsutism, acne, and alopecia. The degree of hirsutism was assessed using the Ferriman-Gallwey scale, and the cutoff point indicative of hirsutism was defined as a score of at least eight points. Biochemical hyperandrogenism involves elevated levels of androgen hormones (testosterone and androstenedione). Pelvic ultrasound was performed using ProSound Alpha 7 equipment (Hitachi-Aloka Medical America Inc., Wallingford, CT, USA) to assess ovarian morphology. The presence of at least 12 circumferentially located follicles that are 2-9 mm in diameter in each ovary or an ovarian volume > 10 ml without the presence of confounding pathology was considered indicative of PCOM. The exclusion criteria were as follows: refusal to participate in the study and diagnosis of hyperandrogenism due to causes other than PCOS, such as nonclassical adrenal hyperplasia, androgen-secreting tumors, or Cushing syndrome. An additional exclusion criterion was the use of oral contraception, glucocorticoids, biguanides, and other drugs, supplements or herbs that could affect the hormonal function and serum androgen levels, for up to six months before participation in the study.

The inclusion criteria for patients with HT included, at least, an elevated serum level of anti-TPO autoantibodies and reduced echogenicity of the thyroid gland on the ultrasound image. This means that all patients had elevated anti-TPO antibodies, and the majority (excluding seven patients) also had elevated anti-Tg antibodies. Echogenicity was assessed in both the thyroid lobes and muscles surrounding the neck. Hypoechogenicity was assessed by comparing the distribution of echoes in the thyroid parenchyma with those in the surrounding neck muscles. For the remaining patients in the study who were not included in the WES, the criterion of PCOM presence may not have been met in cases of PCOS, and an elevated level of anti-Tg autoantibodies, in addition to the hypoechogenic thyroid gland, was sufficient for the diagnosis of HT. Approximately 55% of the patients with HT or PCOS+HT were supplemented with levothyroxine because of additionally diagnosed clinical or subclinical hypothyroidism. At the time of recruitment and blood collection, all patients with HT were euthyroid, either because hypothyroidism had not yet developed or as a result of adequate supplementation. Baseline serum levels of relevant autoantibodies, hormones, and endocrine parameters were determined as part of routine diagnostic procedures. See **Supplementary Table S1** for the ranges of standard concentrations. The demographic and clinical characteristics of the enrolled patients and controls are shown in **Table 1**.

**Table 1:** Clinical and hormonal characteristics of study participants.

Parameter	PCOS *N* = 203	HT *N* = 164	PCOS+HT *N* = 166	Control *N =* 38	Kruskall Wallis
	Median (IQR)	Median (IQR)	Median (IQR)	Median (IQR)	*p*_adj_-value
Age (years)	24 (7)^*^	33 (8)^**^	27 (7.25)	30.5 (10.25)^*^	2.2 × 10^-25^
BMI (kg/m^2^)	25 (7.9)	24 (5)^*^	26 (10.5)	22.1 (4.18)^*^	3.4 × 10^-3^
FSH (mIU/ml)	4.97 (1.67)	5.5 (2.38)^*^	4.75 (2.1)	5.52 (1.23)	2.8 × 10^-4^
LH (mIU/ml)	7.6 (5.53)^*^	4.9 (2.9)^**^	6.06 (4.46)	4.77 (1.39)^*^	3.9 × 10^-13^
LH/FSH	1.5 (1.14)^*^	0.85 (0.44)^**^	1.3 (0.97)	0.81 (0.38)^**^	8.5 × 10^-24^
E_2_ (pg/ml)	37.5 (21.0)^*^	42 (43)	41 (24.5)	41.5 (28)	7.0 × 10^-3^
T (ng/ml)	0.55 (0.22)	0.32 (0.11)^**^	0.56 (0.23)	0.3 (0.1)^**^	1.1 × 10^-50^
A (ng/ml)	4.08 (1.75)	1.9 (0.8)^**^	3.9 (1.37)	1.94 (0.31)^**^	1.6 × 10^-67^
PRG (ng/ml)	0.27 (0.2)^**^	8 (8.48)^**^	0.43 (2.42)	11 (5)^**^	1.6 × 10^-45^
17-OH-PRG (ng/ml)	1.74 (1.34)^*^	1.04 (0.67)^**^	1.38 (1.2)	1.2 (0.57)	7.1 × 10^-18^
DHEAS (μmol/l)	9.26 (4.82)	6.23 (3.48)^**^	8.36 (4.65)	6.88 (3.16)^*^	1.1 × 10^-13^
TSH (μIU/ml)	1.55 (0.83)	1.47 (1.07)^*^	1.72 (1.18)	1.48 (1.12)^*^	5.4 × 10^-2^
fT_4_ (pmol/ml)	12.41 (1.91)	12.9 (2.55)	12.7 (2.38)	12.49 (1.26)	1.9 × 10^-2^
TPO-Ab (IU/ml)	0.33 (0.49)^**^	254 (557)	127 (404)	0.46 (0.64)^**^	8.1 × 10^-76^
Tg-Ab (IU/ml)	1.38 (1.17)^**^	36 (90.58)	41 (95.75)	1 (0.56)^**^	3.6 × 10^-74^

Data are shown as median and interquartile range (IQR). Bold value indicates statistically significant difference with PCOS+HT group (^*^ *p* < 5 × 10^-2^, ^**^ *p* < 10^-4^). A, androstenedione; BMI, body mass index; DHEAS, dehydroepiandrosterone sulfate; E_2_, estradiol; FSH, follicle-stimulating hormone; fT_4_, free thyroxine; HT, Hashimoto’s thyroiditis; LH, luteinizing hormone; PCOS, polycystic ovary syndrome; PRG, progesterone; T, testosterone; Tg-Ab, anti-thyroglobulin antibody; TPO-Ab, anti-thyroid peroxidase antibody; TSH, thyroid-stimulating hormone.

### 2.3 Nucleic acid extraction

Total genomic DNA was extracted from whole blood treated with EDTA using a QIAamp DNA Mini Blood Kit (Qiagen GmbH, Hilden, Germany) according to the manufacturer’s instructions. Quantity and purity of extracted DNA were measured using Qubit™ dsDNA HS Assay Kit on Qubit fluorometer 2.0 (Thermo Fisher Scientific, Waltham, MA, USA) and NanoDrop 2000 spectrophotometer (Thermo Fisher Scientific, Waltham, MA, USA), respectively.

### 2.4 Whole-exome sequencing and exome sequence data analysis

Exome regions were captured using the Twist Human Core Exome Kit with RefSeq and Mitochondrial Panel (Twist Bioscience, San Francisco, CA, USA). Paired-end sequencing (2 × 100 bp) was performed using an Illumina NovaSeq 6000 next-generation sequencing platform (San Diego, CA, USA), yielding a minimum read depth of 40x in the cohort. The generated reads were aligned to the human reference genome build, hg19, from the UCSC Genome Browser, using Bowtie2 (version 2.4.4) (50). After removing duplicate reads and base quality recalibration using Picard tools (http://broadinstitute.github.io/picard), genetic variations were determined using the Genome Analysis Toolkit (GATK) HaplotypeCaller with the default parameters (51). Variant genotyping was implemented using the GATK GenotypeGVCFs tool with the dbSNP build 155 vcf file as a reference and the variant call confidence score set to 50.

After variant quality control (QC), ethnicity was inferred using the R package EthSEQ (version 2.1.4) (52) based on ancestry reference samples from the 1000 Genomes Project (http://www.1000genomes.org), indicating that all samples were within the European population. Principal Component Analysis of WES data generated for all 250 participants, implemented using Plink version 1.9, identified seven outliers that were excluded from further analysis. A Variant Effect Predictor (VEP; GRCh37 Ensembl release version 105) (53) was used to assess the potentially deleterious effects (high, moderate, low, or modifier impact) of the exome variants (http://www.ensembl.org/index.html).

### 2.5 Association analyses

Four association analyses were conducted using a variant in the hg19 sequence as the reference allele. The principal analysis assumed a dominant mode of inheritance, where the number of alternate allele genotypes and reference allele homozygotes were compared using Fisher’s exact test conducted in rvtests version 20170613 (54). The same test was used for two other analyses: a comparison of the number of alternate allele homozygotes with other genotypes (recessive mode of inheritance) and a comparison of both alternate and reference allele frequencies (allelic test). Individual genotype frequencies were analyzed using Plink version 1.9.

Functional enrichment analysis in sets of selected genes was conducted using the Cytoscape platform v.3.6.1 with the ClueGO v.2.5.1 application (55) and GeneAnalytics program (geneanalytics.genecards.org) (56), based on Gene Ontology (GO) and Kyoto Encyclopedia of Genes and Genomes (KEGG) databases, using the default settings and the *p*-value adjusted (*p_adj_*-value) for multiple comparisons with the Benjamini–Hochberg algorithm with significance threshold < 0.05 (57). Candidate gene prioritization was conducted using the Endeavour approach (https://endeavour.esat.kuleuven.be/) (58), using genes associated with HT and PCOS according to the Malacards database (59) as the training set.

### 2.6 Prediction models – stepwise forward logistic regression analysis

Multivariate logistic regression was used to create models to predict the risk of joint occurrence of PCOS and HT. For the selection of marker SNPs, variants significantly associated (*p* < 0.05) with the joint occurrence of PCOS and HT in any of the four association analyses were clumped using Plink version 1.9 with default parameters (variants with *p* < 10^-3^ in the 250 kb region). Body mass index (BMI) value was included as an additional variable. Predictive analysis was performed using a stepwise forward logistic regression method, with the Akaike information criterion (AIC) as a variable selection criterion and the step function of the R basic statistics package. Significant SNPs were ranked according to their AIC values, starting from the variant with the lowest AIC value, and were sequentially introduced into the prediction model. Nagelkerke’s pseudo-*R^2^* value for each step was computed with the DescTools package, version 0.99.40 (60) to estimate the proportion of the overall risk of developing PCOS and HT.

The area under the ROC curve (AUC) value, describing the accuracy of the prediction, was computed using the pROC package, version 1.16.2 (61). 10-fold cross-validation was performed to avoid model overfitting. The remaining parameters describing the prediction accuracy were calculated using two probability threshold levels: 50% and 70%. A probability threshold of 50% means that PCOS+HT was predicted if the prediction probability was ≥ 50%. If the probability was < 50%, a non-PCOS+HT stage was predicted. A probability threshold of 70% means that PCOS+HT was predicted only if the prediction probability was ≥ 70%. Prediction results that did not reach the 70% probability threshold were considered to be inconclusive (30-70%) or no-PCOS+HT (< 30%).

### 2.7 Monte Carlo feature selection and interdependencies discovery – feature importance

The Monte Carlo feature selection and interdependency discovery (MCFS-ID) method implemented in the rmcfs R package, as the mcfs() function, was used to perform supervised feature selection (62, 63). The data input to the mcfs() function was in the form of a decision table, in which features were stored as columns, and one row reflected one patient. To fix the input data values, column names, and attribute types, the fix.data() function was applied (63). The initial data consisted of 175,376 features (WES-identified variants and BMI). Of these, 1979 showed no variance across patients and was excluded. To evaluate the feature potential for distinguishing patients between groups (P+H *vs.* P and P+H *vs.* H), two computational runs were performed using the MCFS-ID algorithm. In each, the algorithm builds thousands of decision trees on randomly selected subsets of features and patients to obtain the relative importance (RI) score for each feature. Based on the calculated RI, two rankings of features that best predicted the samples belonging to a given group were returned. The permutation method was used to evaluate the significance of the top features. Because further steps of the analysis assumed verification of the significance of the returned features, the relaxed cutoff point was used according to the critical angle cutoff point implemented in the MCFS-ID. Finally, to maintain an equal number of features, the top 15 variants were selected from each comparison and the ID module of the mcfs() function was used to reveal the associations between these variants and their most important 40 interactions. The created networks of interdependencies are presented in the form of directed SVG graphs.

### 2.8 TaqMan genotyping and data analysis

To further verify the association of the selected SNPs, DNA samples were genotyped using TaqMan SNP Genotyping Assays (Thermo Fisher Scientific, Waltham, MA, USA), Sensi FAST Probe Hi-ROX Kit (Bioline Ltd., London, United Kingdom), and 7900HT Real-Time PCR system (Thermo Fisher Scientific, Waltham, MA, USA) in a 384-well format. The Hardy–Weinberg equilibrium concordance of SNPs selected for verification was tested using the HardyWeinberg R package version 1.7.5 (64). Differences in allele frequencies between groups were verified using Fisher’s exact test (implemented in the EpiTools R package), whereas differences in genotype frequencies were assessed using the chi-squared test. The *p*-value significance threshold was adjusted using the Benjamini–Hochberg false discovery rate (FDR) algorithm (57). Odds ratios (ORs) and 95% confidence intervals (CIs) were estimated using the normal approximation implemented in the EpiTools R package, version 0.5–10 (65). Unless otherwise noted, OR values were calculated using the hg19 sequence allele as a reference.

## 3 Results

In this study, WES was used to identify genetic variants associated with the joint occurrence of PCOS and HT. A total of 571 women were included in the study: 203 with PCOS, 164 with HT, 166 with both disorders, and 38 healthy controls. All the patients were of European ancestry. **Table 1** presents the characteristics of the study participants. Patients with HT were more likely (*p* < 10^-4^) to have lower serum levels of luteinizing hormone (LH), dehydroepiandrosterone (DHEAS), androstenedione, testosterone, 17-OH-PRG, and LH/follicle-stimulating hormone (FSH) ratio and higher levels of PRG than patients with combined PCOS and HT. Whereas, patients with PCOS had lower serum levels of anti-TPO and anti-Tg antibodies and PRG than patients with both disorders. For the WES, 250 study participants were included: 70 patients with PCOS, 71 patients with HT, 71 patients with both PCOS and HT, and 38 control individuals.

### 3.1 Association analyses

After QC filtering of the exome sequence data, 175,374 qualifying variants located in 18,886 genes and 98 regulatory regions were selected for further association analyses (median 25,603 variants per sample). Of these, 46% were missense variants, 35% were synonymous variants, and 7% were intronic variants according to VEP (GRCh37 Ensembl). Association analyses, assuming a dominant model of inheritance, were performed for the following group comparisons: P+H *vs.* H, P+H *vs.* P, H *vs.* P, P+H *vs.* K, H *vs.* K, and P *vs*. K. The number of significantly differentiating variants (*p* < 0.05) in these comparisons was 2061, 1960, 1583, 2275, 2381, 2558, respectively, including 20, 31, 18, 26, 16, 30 with *p* < 10^-3^, respectively, and 7, 19, 7, 10, 7, 17 with *p* < 5×10^-4^, respectively. The most significant association with co-occurrence of PCOS and HT was observed for rs17855988 in *ELN* gene (*p* = 4.96 × 10^-6^), unknown variant 9:139846575_A/AGGTG located in the regulatory region upstream of the *LCN12* (*p* = 4.3 × 10^-5^) and rs4758289 in *TUB* (*p* = 7.65 × 10^-5^) in P+H *vs.* P; rs7259 in *CERCAM* (*p* = 1.95 × 10^-4^) and rs562859 in *OPRM1* (*p* = 2.99 × 10^-4^) in P+H *vs.* H; rs4746970 in *TYSND1* (*p* = 6.2 × 10^-5^) and rs7145565 in *CDCA4* (*p* = 1.71 × 10^-4^) in P+H *vs.* K comparison.

### 3.2 Common or unique variants and functional enrichment analyses

**Figure 1:**
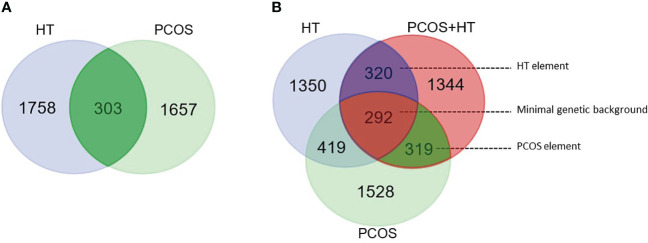
Venn diagram for significantly differentiating variants. **(A)** Pairwise comparisons of combined PCOS and HT (PCOS+HT) with solely PCOS or HT groups. A total of 303 variants were common for both comparisons. **(B)** Pairwise comparisons of PCOS+HT, PCOS, and HT groups with the control (K) group. 293 variants were common for all three comparisons (‘minimal genetic background’), whereas, 319 and 320 variants were common for PCOS+HT and PCOS (‘PCOS element’), and for PCOS+HT and HT (‘HT element’) comparisons with the K group, respectively.

**Figure 2:**
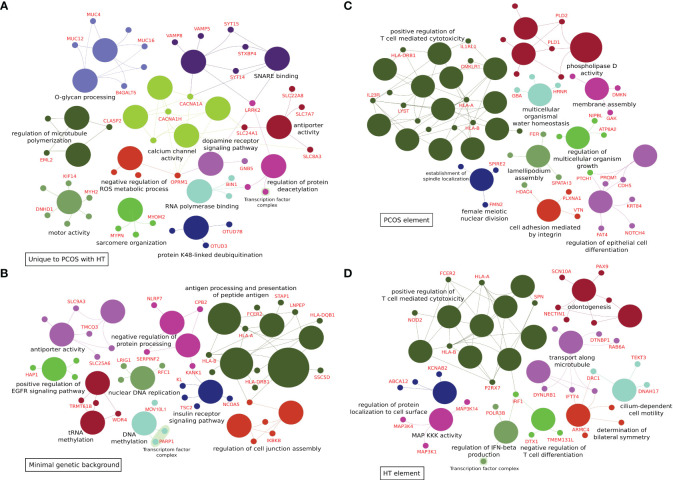
ClueGo analyses of functional enrichment in biological processes and molecular function Gene Ontology terms. **(A)** 303 variants differentiating combined PCOS and HT (PCOS+HT) from both PCOS and HT groups (common for P+H *vs.* H and P+H *vs.* P comparisons). **(B)** 292 variants differentiating all three groups of patients (PCOS+HT, PCOS, and HT) and control (K) group – assigned as ‘minimal genetic background’. **(C)** 319 variants differentiating PCOS+HT and PCOS groups from K group (common for P+H *vs.* K and P *vs.* K comparisons) – assigned as ‘PCOS element’. **(D)** 320 variants differentiating PCOS+HT and HT groups from K group (common for P+H *vs.* K and H *vs.* K comparisons) – assigned as ‘HT element’.

Venn diagrams were created to identify unique or common variants, differentiating patients with both disorders from those with PCOS or HT alone. As shown in **Figure 1A**, 303 variants were unique to PCOS with HT, as they differentiated the double-case group from both single-case groups (common for P+H *vs.* H and P+H *vs.* P comparisons). According to the VEP, 132 of these variants were low-impact (mostly synonymous), 115 were moderate-impact, four were high-impact, and 52 were changes in non-coding regulatory sequences (modifier impact) (**Supplementary Table S2**). To increase the chance of detecting biological processes related to the above genetic variants, all variants, regardless of the expected effect, were included in functional enrichment analyses based on the GO and KEGG databases. Analysis of 234 genes to which the above 303 variants were assigned indicated a significant association (*p_adj_* < 0.05) with 40 biological processes and signaling pathways, such as *Protein O-linked glycosylation* and *O-glycan processing*, *Voltage-gated calcium channel activity* and *Calcium ion transmembrane transporter activity, Regulation of microtubule polymerization and depolymerization* and *Motor activity* (**Figure 2A**; **Supplementary Table S3**).

A Venn diagram of the results of patient group comparisons with the control group indicated 292 common variants (assigned as ‘minimal genetic background’), differentiating all three groups of patients (P+H, P, and H) from the K group (**Figure 1B**). Among them, 123 were variants with low impact, 113 with moderate, six with high impact, and 50 variants were potential modifiers (**Supplementary Table S4**). In total, 88 GO and KEGG terms were significantly associated with the 212 genes assigned to these variants, including *Antigen processing and presentation of peptide antigen*, *Regulation of insulin receptor signaling pathway, Vesicular transport between the endoplasmic reticulum and the Golgi apparatus*, *Dynein light intermediate chain binding, Regulation of cell junction assembly*, *Peroxisome organization* and *Positive regulation of epidermal growth factor receptor signaling pathway* (**Figure 2B**; **Supplementary Table S5**).

The same Venn diagram (**Figure 1B**) indicated 319 variants common for only the P+H and P groups in comparison with the K group (assigned as ‘PCOS element’). Of these, 126 were low-impact variants, 144 were moderate-impact, five were high-impact, and 44 were located in the regulatory regions (**Supplementary Table S6**). Functional enrichment analysis of 216 genes assigned to these variants indicated their involvement in 95 biological processes and molecular activities such as *Regulation of T cell-mediated cytotoxicity* and *Immunoglobulin mediated immune response*, *Female meiotic nuclear division* and *Spindle localization*, *Lamellipodium assembly* and *Cell adhesion*, *Phospholipase D signaling pathway* and *Choline metabolism* (**Figure 2C**; **Supplementary Table S7**).

Similarly, 320 variants were common only for P+H *vs.* K and H *vs*. K comparisons (assigned as ‘HT element’) (**Figure 1B**). Low impact was attributed to 116 variants, moderate impact to 140 variants, high impact to four variants, and modifier impact to 60 variants (**Supplementary Table S8**). The 244 genes assigned to these variants were related to 87 GO and KEGG terms, including *Regulation of T cell-mediated cytotoxicity and immunity*, *Interferon-gamma-mediated signaling pathway*, *Cilium or flagellum-dependent cell motility* and *Axoneme localization*, *Bilateral symmetry determination*, *Microtubule-based transport* and *Cell adhesion molecules* (**Figure 2D**; **Supplementary Table S9**).

### 3.3 Prediction modeling based on WES data - stepwise forward logistic regression

Considering the different inheritance modes of the genetic variants, three additional association analyses were conducted: comparison of the number of alternate allele homozygotes with other genotypes (recessive mode of inheritance), comparison of both alternate and reference allele frequencies (allelic test), and comparison of individual genotype frequencies (genotype test). Statistically significant variants (*p* < 0.05) in any of the four association analyses were subjected to a clumping procedure to select marker SNPs (*p* < 10^-3^ in the 250 kb region). Stepwise forward logistic regression was used to create models predicting the risk of the joint occurrence of PCOS and HT, using the AIC minimization approach for significant variable selection. The selected variants were ranked according to their AIC values and were sequentially introduced into the prediction model. Two prediction models were created: differentiating PCOS+HT from PCOS (PCOS model) and differentiating PCOS+HT from HT (HT model).

As estimated using Nagelkerke’s pseudo-*R^2^* statistics, the eight variants included in the PCOS model explained 83% of the overall variation between patients with combined PCOS and HT and those with PCOS alone (**Table 2**). The model prediction accuracy expressed by the AUC was 0.912 (95% CI 0.861-0.956) with a sensitivity of 88.7% and a specificity of 92.9%. The accuracy of the model for the 10-fold cross-validation was 0.84 ± 0.12. Seven variants were selected for the HT model, which explained 73% of the overall variation between the co-occurrence of both diseases and HT alone (**Table 2**). The AUC value of this model was 0.874 (95% CI 0.819-0.929), sensitivity 88.7%, specificity 85.9%, and accuracy for 10-fold cross-validation 0.84 ± 0.08. The BMI was not selected for any of the models.

**Table 2:** The results of the stepwise selection for the logistic regression models based on WES data.

Model differentiating PCOS+HT and PCOS					
dbSNP ID ^a^	Gene	AIC ^b^	AIC change (%)	*R* ^2 c^	*R* ^2^ change (%)
9:139846575_A/AGGTG, (rs11787588) ^d^	*intergenic*	183.50		0.16	
rs5751516 ^e^	*IGLV2-23*	167.44	16.06 (8.8)	0.32	0.16 (98)
rs61750041	*CERKL*	152.16	15.28 (9.1)	0.45	0.13 (40.6)
rs59506446, (rs7129499) ^d^	*KRTAP5-2*	136.62	15.54 (10.2)	0.56	0.11 (24.4)
rs2777962	*FCRL4*	123.73	12.89 (9.4)	0.65	0.09 (16.1)
rs7427	*MSRB2*	113.26	10.47 (8.5)	0.71	0.07 (10.8)
rs10789501 ^e^	*CYP4A22*	101.18	12.08 (10.7)	0.78	0.07 (9.9)
rs7499814 ^e^	*BANP*	91.30	9.88 (9.8)	0.83	0.04 (5.1)
Model differentiating PCOS+HT and HT					
dbSNP ID ^a^	Gene	AIC ^b^	AIC change (%)	*R* ^2 c^	*R* ^2^ change(%)
rs3737075 ^e^	*FAM207A*	184.38		0.16	
rs812847	*NWD1*	168.49	15.89 (8.6)	0.32	0.15 (93.8)
rs675026, (rs677830) ^d^	*OPRM1*	155.46	13.03 (7.7)	0.43	0.12 (37.5)
rs11642122	*intergenic*	143.10	12.36 (8.0)	0.52	0.09 (20.9)
rs3818222	*BPIFA3*	131.95	11.15 (7.8)	0.60	0.08 (15.4)
rs2075577	*UCP3*	122.76	9.19 (7.0)	0.67	0.07 (11.7)
rs8066132	*SLFNI2L*	113.17	9.59 (7.8)	0.73	0.06 (9.0)

The marker variants (*p* < 10^-3^), ranked using the Akaike Information Criterion (AIC), were sequentially implemented into the model, starting with the variant with the lowest AIC value.

^a/^SNP identifier based on NCBI SNP database (http://www.ncbi.nlm.nih.gov/SNP/).

^b/^AIC value calculated after sequential implementation of the ranked SNPs.

^c/^Nagelkerke pseudo-*R* ^2^ value calculated after sequential implementation of the ranked SNPs.

^d/^The probe was unavailable; SNP located in the same 250 kb clumping region was chosen (ID in the parenthesis).

^e/^The probe was unavailable; there were no other SNPs in the clumping region.

### 3.4 Importance of the features – Monte Carlo feature selection and interdependencies discovery

The MCFS-ID algorithm was used to select the most informative features (variants) and rank them according to the calculated RI values. Analyses were performed on the WES-identified variants to compare the P+H group with the P or H groups, yielding ranking lists of variants that best distinguished patients with co-occurrence of PCOS and HT from those with either PCOS or HT alone. Among the variants with the highest RI values, rs36104512 in *RANBP17* was common in both comparisons (P+H *vs.* P and P+H *vs.* H). In addition, the best-differentiating PCOS+HT and PCOS groups were rs17855988 in *ELN*, rs1061638 in *AHSA1*, rs140634372 in *PRDM5*, and rs77570237 in *IGSF9*. The variants that best differentiated the PCOS+HT and HT groups were rs62638683 in *GPR37*, rs185466872 in *MYO18B*, rs1043424 in *PINK1*, and the undescribed 19:6751068_T/A variant in *TRIP10* (**Supplementary Figure S1**). BMI was not ranked among the top 500 features in any comparison.

**Figure 3:**
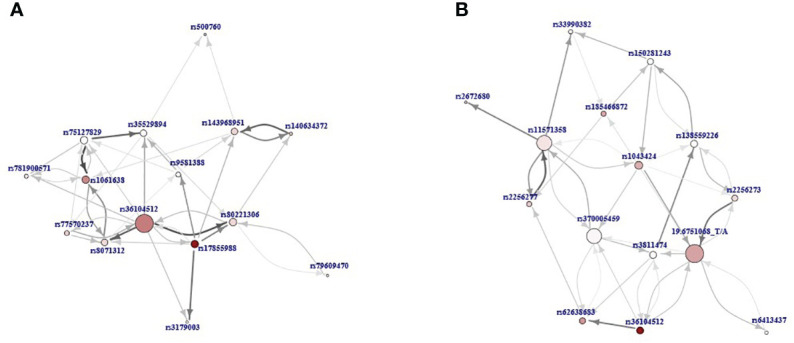
The directed graphs presenting interdependencies of the top 15 variants according to the relative importance (RI) value and their 40 most important interactions: **(A)** P+H *vs.* P, **(B)** P+H *vs.* H. Nodes represent variants and the thickness of the edges shows the importance of the association. The direction of the arrow points from the feature that was closer to the root in the decision trees to the feature further away. The size of the node reflects the number of interdependencies (the more interdependencies the larger node) and its color intensity represents the RI value (the more intensive, the higher value).

For the top 15 variants, selected based on the adopted critical angle cutoff point, and for their 40 most significant interactions, a network of interdependencies was built and presented as a directed SVG graph, where the nodes represent variants, and the thickness of the edges shows the importance of the association (**Figure 3**). Thus, features that do not have significant predictive power in themselves (e.g., due to very low prevalence in the population) in the presence of other features become significant for differentiating patients between groups and can be considered potentially causal. Two hub nodes, rs36104512 and 19:6751068_T/A, had the largest number of associations with other variants in the P+H *vs.* P and P+H *vs.* H analyses, respectively. Interestingly, in the P+H *vs.* P graph (**Figure 3A**), there were fewer edges with arrows in both directions between pairs of variants, indicating a stronger hierarchical relationship between them than between pairs of features in the P+H *vs.* H.

### 3.5 TaqMan genotyping

Several approaches have been applied to select differentiating variants for further validation by TaqMan DNA genotyping: (1) association analyses and Venn diagram selection of unique or common variants significant in pairwise comparisons, (2) stepwise forward logistic regression selection for prediction models, (3) MCFS-ID selection of feature importance, and (4) candidate gene prioritization using the Endeavour approach. Some of the differentiating variants could not be selected for genotyping validation because TaqMan probes for these variants were not commercially available, and universal custom probes or primers could not be designed because of the architecture of variant locations. Finally, 77 SNPs were selected for allele frequency analyses in 533 patients: 203 with PCOS, 164 with HT, and 166 with both disorders. The estimated variant OR, 95% CI, and significance *p*-value in P+H *vs.* P and P+H *vs.* H comparisons of allele and genotype frequencies are presented in **Supplementary Table S10**. Six and three SNP associations were significant in the allelic test with FDR *p_adj_*-value < 0.05 for P+H *vs.* P and P+H *vs.* H, respectively (**Table 3**). Additionally, the two variants were significantly associated (*p_adj_ <* 0.05) with the combined PCOS and HT in the P+H *vs.* P genotype frequency comparison. The most significant differentiation between PCOS+HT and PCOS groups was observed for rs4484951 in *FNDC7* gene (OR 4.62; 95% CI 1.95-12.85) and between PCOS+HT and HT groups for rs8101480 in *HIF3A* gene (OR 5.88; 95% CI 1.89-25.0). Two significant SNPs, rs4955418 in *CCDC71* and rs28364969 in *CDK20*, were common in both comparisons.

**Table 3:** TaqMan genotyping significant associations.

dbSNP_ID ^a^	Gene	MA	MAF	PCOS+HT *vs.* PCOS			PCOS+HT *vs*. HT		
				OR allelic (95% CI)	Allelic *p*_adj_	Genotype *p*_adj_	OR allelic (95% CI)	Allelic *p*_adj_	Genotype *p*_adj_
rs1061638	*AHSA1*	A	0.33	1.08 (0.78-1.49)	0.736	**0.009**	1.15 (0.82-1.63)	0.559	0.451
rs4955418	*CCDC71*	A	0.35	0.59 (0.43-0.81)	**0.026**	**0.046**	0.51 (0.36-0.71)	**0.005**	**0.014**
rs28364969	*CDK20*	T	0.34	0.56 (0.40-0.79)	**0.026**	**0.023**	0.57 (0.40-0.76)	**0.046**	0.081
rs4484951	*FNDC7*	C	0.02	4.62 (1.95-12.9)	**0.026**	**0.021**	3.76 (1.59-10.5)	0.059	0.081
rs4656077	*GBP3*	A	0.33	0.64 (0.48-0.86)	**0.048**	**0.047**	0.69 (0.51-0.94)	0.111	0.171
rs634710	*GOLGA1*	A	0.43	1.47 (1.10-1.97)	0.085	**0.047**	1.34 (0.99-1.83)	0.173	0.081
rs1007298	*GUSBP11*	T	0.32	1.69 (1.23-2.33)	**0.026**	**0.049**	1.59 (1.15-2.22)	0.077	0.140
rs8101480	*HIF3A*	T	0.13	1.45 (0.70-2.99)	0.490	0.431	5.88 (1.89-25.0)	**0.046**	**0.039**
rs41298115	*RAB6A*	C	0.01	NA	**0.042**	**0.047**	NA	0.404	0.395

Bold indicates genotype-phenotype relationship significant at FRD *p*-adjusted (*p*_adj_) < 0.05. MA; minor allele, MAF; MA frequency, CI; confidence interval, OR; odds ratio.

^a/^SNP identifier based on NCBI SNP database (http://www.ncbi.nlm.nih.gov/SNP/).

### 3.6 Validation of the WES prediction models

Based on the TaqMan genotyping results, both prediction models built on WES data were further validated in an unrelated group of 322 patients: 133 with PCOS, 93 with HT, and 96 with both disorders. However, TaqMan probes were available for three of the eight variants included in the original PCOS model, and two additional probes were selected for variants from the same clamping region as the marker SNP (**Table 2**). The overall accuracy of the prediction of the joint occurrence of PCOS and HT based on these five variants was as follows: AUC, 0.536 (95% CI 0.437-0.635); sensitivity, 14.7%; specificity, 88.6%; 10-fold cross-validation accuracy 0.54 ± 0.05. In total, they explained only about 2.4% of the total variation in PCOS+HT. In the case of the WES data-based HT model, probes for five of the seven variants were available, and an additional one was selected for the variant from the same clumping region (**Table 2**). The calculated accuracy of model prediction expressed by AUC was 0.594 (95% CI 0.523-0.665), with the sensitivity of 63.8% and the specificity 54.8%, and the 10-fold cross-validation accuracy 0.53 ± 0.06. The selected variants accounted for a total of 8.3% the variation in PCOS+HT.

### 3.7 Prediction modeling based on TaqMan genotyping data - stepwise forward logistic regression

To create more accurate and useful models for predicting the risk of the joint occurrence of PCOS and HT, stepwise forward logistic regression was used to select variants from a set of 77 differentiating SNPs based on genotyping results in a group of 533 patients. In the analysis, the FAM homozygote was used as the reference. When absent, the FAM/VIC genotype was used as the reference. The final PCOS (differentiating PCOS+HT and PCOS) and HT (differentiating PCOS+HT and HT) models are presented in **Table 4**. The variants selected for the models accounted for 47.4% and 44.3% of the PCOS+HT variability compared with the PCOS and HT groups, respectively.

**Table 4:** The results of the stepwise selection for the logistic regression models based on TaqMan genotyping data.

Model differentiating PCOS+HT and PCOS					
dbSNP ID ^a^	Gene	AIC ^b^	AIC change (%)	*R* ^2 c^	*R* ^2^ change (%)
rs4484951	*FNDC7*	384.80		0.048	
rs41298115	*RAB6A*	375.52	9.28 (2.4)	0.098	0.050 (102.8)
rs634710	*GOLGA1*	365.39	10.13 (2.7)	0.157	0.059 (60.8)
rs11787588	*PRR31*	358.94	6.45 (1.8)	0.199	0.042 (26.8)
rs3179003	*NCR3*	353.30	5.64 (1.6)	0.229	0.030 (15,0)
rs17301182	*DYNC2H1*	347.47	5.83 (1.7)	0.267	0.037 (16.3)
rs1061638	*AHSA1*	342.59	4.88 (1.4)	0.299	0.033 (12.2)
rs4656077	*GBP3*	338.48	4.11 (1.2)	0.328	0.029 (9.7)
rs8066132	*SLFN12L*	333.61	4.87 (1.4)	0.359	0.031 (9.4)
rs12038198	*CGN*	329.79	3.82 (1.1)	0.379	0.020 (5.5)
rs276937	*DSC3*	326.64	3.15 (1.0)	0.402	0.024 (6.2)
rs28364969	*CDK20*	322.77	3.87 (1.2)	0.428	0.025 (6.3)
rs1786263	*CEP192*	320.93	1.84 (0.6)	0.446	0.018 (4.3)
rs79609470	*MICALCL*	319.18	1.75 (0.5)	0.458	0.012 (2.6)
rs1063479	*PLEK*	317.90	1.28 (0.4)	0.474	0.016 (3.5)
Model differentiating PCOS+HT and HT					
dbSNP ID ^a^	Gene	AIC ^b^	AIC change (%)	*R* ^2 c^	*R* ^2^ change (%)
rs4484951	*FNDC7*	344.56		0.056	
rs8101480	*HIF3A*	336.39	8.17 (2.4)	0.106	0.050 (89.9)
rs3829817	*FBN3*	331.13	5.26 (1.6)	0.150	0.044 (41.4)
rs2256273	*FAM189A1*	326.18	4.95 (1.5)	0.191	0.041 (27.3)
rs4955418	*CCDC71*	322.61	3.57 (1.1)	0.224	0.034 (17.6)
rs11564148	*LRRK2*	318.92	3.69 (1.1)	0.257	0.033 (14.7)
rs12038198	*CGN*	316.99	1.93 (0.6)	0.274	0.017 (6.4)
rs909920	*TPSG1*	315.35	1.64 (0.5)	0.297	0.023 (8.5)
rs28364969	*CDK20*	313.13	2.22 (0.7)	0.322	0.025 (8.4)
rs1054645	*CACNA1H*	310.35	2.78 (0.9)	0.349	0.027 (8.3)
rs41275060	*EFNB2*	308.97	1.38 (0.4)	0.369	0.021 (5.9)
rs1156287	*STXBP4*	308.08	0.89 (0.3)	0.388	0.018 (5.0)
rs595844	*AZU1*	306.40	1.68 (0.5)	0.409	0.021 (5.4)
rs1800472	*TGFB1*	304.14	2.26 (0.7)	0.424	0.015 (3.8)
rs634710	*GOLGA1*	302.78	1.36 (0.4)	0.443	0.019 (4.5)

Selected SNPs, ranked using the Akaike Information Criterion (AIC), were sequentially implemented into the model, starting with the variant with the lowest AIC value.

^a/^SNP identifier based on NCBI SNP database (http://www.ncbi.nlm.nih.gov/SNP/).

^b/^AIC value calculated after sequential implementation of the ranked SNPs.

^c/^Nagelkerke pseudo-*R* ^2^ value calculated after sequential implementation of the ranked SNPs.

The prediction accuracy parameters were calculated for both models (**Table 5**). The AUC value for the PCOS model was 0.785 (95% CI 0.731-0.831), sensitivity was 85.7%, specificity was 68.1%, and the 10-fold cross-validation accuracy was 0.71 ± 0.06. The AUC value for the HT model was 0.776 (95% CI 0.724-0.828), sensitivity was 72.4%, specificity was 82%, and 10-fold cross-validation accuracy was 0.71 ± 0.12. When a 70% probability threshold was applied, the accuracy of both models prediction improved, increasing the AUC value to approximately 0.87 and the specificity of the prediction to 93% (**Table 5**). However, inconclusive results were obtained at 35% and 40% for PCOS and HT models, respectively.

**Table 5:** The prediction parameters of models differentiating PCOS+HT and PCOS (PCOS model) or HT (HT model).

Prediction parameters	Probability threshold	PCOS model [95% CI]	HT model [95% CI]
AUC	50%	0.785 [0.731-0.831]	0.776 [0.724-0.828]
Sensitivity		85.7%	72.4%
Specificity		68.1%	82.0%
Accuracy		0.708 [SD 0.056]	0.711 [SD 0.120]
AUC	70%	0.875 [0.824-0.927]	0.865 [0.811-0.919]
Sensitivity		77.6%	76.8%
Specificity		93.4%	92.9%
Inconclusive		35%	40%

Accuracy; 10-fold cross validation accuracy, AUC, area under the ROC curve; CI, confidence interval; SD, standard deviation.

## 4 Discussion

Here, for the first time, WES was used to explore the possibility of predicting the joint occurrence of PCOS and HT. In addition, our study is among the largest WES analyses of any of these diseases (42–49). Despite the high heritability of PCOS and HT as indicated by familial clustering and twin studies (32, 66), the mode of inheritance remains unclear. The lack of clear genetic causes reinforces the hypothesis of the polygenic nature and genetic heterogeneity of both diseases. Therefore, in our analyses, we considered different modes of disease inheritance. Additionally, the analyses included both common and rare variants with different predicted impacts, increasing the chances of identifying biological significance and putative associations with the joint occurrence of the two diseases.

### 4.1 Association and functional enrichment analyses

One of the main aims of this study was to identify variants that are unique or common to the double-case disease compared to PCOS or HT alone and to determine the biological significance of the genes assigned to these variants. An important observation from functional enrichment analyses was that variants differentiating PCOS+HT from PCOS and/or HT were assigned to mostly similar or closely related functional pathways, even if they were unique for a given comparison. This suggests that both diseases share a large portion of their genetic background, and the joint occurrence of both diseases may be determined by the emergence of additional variants that disrupt the same basic processes and are mutually predisposed to the second disease.

Strikingly, several of the same GO BP terms related to the category of immune response were significantly enriched with genes assigned to two otherwise non-overlapping sets of variants, ‘PCOS element’ and ‘HT element’, such as *Interferon-gamma-mediated signaling pathway*, R*egulation of lymphocyte- and T cell-mediated cytotoxicity and immunity*, *Positive regulation of cell killing*, *Positive regulation of adaptive immune response*, *Positive regulation of adaptive immune response based on somatic recombination of immune receptors built from immunoglobulin superfamily domains* and *Detection of other organism and biotic stimulus* (**Supplementary Tables S7**, **S9**). In addition, among the significant GO categories characterizing the set of variants comprising the ‘minimal genetic background’, several were related to processes of antigen processing and peptide antigen presentation via Major histocompatibility complex (MHC) class I (**Supplementary Table S5**), that are prerequisites for activation of the adaptive immune system. Antigen molecules are expressed on the surface of target cells and presented in association with MHC class I or II molecules, typically to CD8+ cytotoxic T (Tc) cells or CD4+ helper T (Th) cells, respectively. The cross-presentation of autoantigens can lead to impaired self-tolerance and the development of autoimmune diseases (67). Overall, this finding may support the concept of an autoimmune etiology of PCOS (68), or more likely, indicate that the systemic immune activation created in PCOS promotes the development of autoimmune diseases, explaining the repeatedly observed association between PCOS and various autoimmune conditions (69, 70).

Interferon-gamma (IFN-γ), a pro-inflammatory cytokine produced by activated T cells, plays an essential role as a mediator of the immune response. It favors the development of Th1 cells over Th2 cells and assists Th1 cells in macrophage activation and B-cell isotype switching (67). Several studies have suggested a protective effect of endogenous IFN-γ in T cell-mediated autoimmune diseases. For example, produced by Th1 cells counteracts the development of Th17 cells, inhibits the proliferation of Th2 but not Th1 cells, and stimulates the suppressive activity of thymus-derived CD4+CD25+ natural regulatory T (Treg) cells (71, 72). Chronic inflammation is a hallmark of both HT and PCOS (73, 74). In patients with HT, the expression of interleukin (IL)-17 was elevated and was significantly correlated with the levels of thyroid hormone, anti-TPO, and anti-Tg antibodies (75). In turn, IFN-γ and IL-10 levels were significantly lower in the HT patients compared with the controls. Similarly, PCOS patients had lower levels of IFN-γ than healthy women, and in the rat model of DHEA-induced PCOS, decreased levels of IFN-γ were observed compared to control rats, which was negatively correlated with elevated testosterone levels (76). DHEA was shown to inhibit the proliferation and promote the apoptosis of ovarian granulosa cells by downregulating the expression of IFN-γ (76).

One of the four unique SNPs that significantly differentiated PCOS+HT and PCOS (*p_adj_* < 0.05) in TaqMan genotyping was rs4656077 in the *GBP3* gene (**Table 3**), which encodes a guanylate-binding protein belonging to the family of IFN-γ-inducible GTPases involved in host defense against infection and inflammasome response, as well as in metabolic inflammatory diseases and cancer (77). This variant was selected to the final prediction model differentiating PCOS+HT and PCOS, as were the SNPs in *NCR3* and *SLFN12L* (**Table 4**). *NCR3* encodes natural cytotoxicity-triggering receptor 3, which interacts with CD247, a T cell receptor (TCR), and activates natural killer cell cytotoxicity and cytokine secretion (78). *SLFN12L* belongs to the Schlafen family of growth-regulating genes involved in thymocyte development and T cell activation. Its upregulation in primary immune cells depends on autocrine type I IFN signaling. SLFN12L has been suggested to play a role in T cell quiescence (79).

The GO biological processes related to *O-*glycan processing, voltage-gated calcium channels, and transporter activity were significantly enriched in genes assigned to variants differentiating PCOS+HT from all three other groups (**Supplementary Table S3**). Glycosylation is one of the most abundant post-translational modifications and glycoproteins play essential roles in diverse processes, including inflammatory and immune responses (80). *O*-glycosylation affects the structure and function of cell surface proteins and modifies antigen processing and presentation to T cells (81). Specific *O-*GlcNAc modifications are important for T cell activation and their blocking reduces IL-2 production and cell proliferation (82). Changes in *O*-glycan composition are associated with different metabolic conditions and disorders, including autoimmune diseases, type II diabetes, and cardiovascular diseases (83, 84). *O*-glycans are present in high concentrations in the zona pellucida surrounding mammalian eggs and play critical roles in fertilization (85). They are involved in sperm-oocyte binding and acrosome reaction induction (86). Oocyte core 1-derived *O*-glycans are involved in the regulation of cumulus-oocyte complex development by modifying the extracellular matrix (ECM) composition (87). *O*-glycan-deficient oocytes exhibit altered follicle development, resulting in the production of more follicles by increasing FSH sensitivity and reducing apoptosis (88).

Voltage-gated Ca^2+^ channels are multi-subunit complexes activated by membrane depolarization that allow Ca^2+^ influx into the cell. In the ovary, intracellular Ca^2+^ plays an essential role in folliculogenesis (89), oocyte maturation, fertilization, and early embryonic development, particularly in egg activation, blocking polyspermy, and egg-to-embryo transition (90). The α1 subunit of the T-type voltage-gated Ca^2+^ channel CaV3.2, encoded by *Cacna1h*, mediates Ca^2+^ influx into mouse oocytes and is required to increase the total and endoplasmic reticulum Ca^2+^ stores in eggs (91). A variant of *CACNA1H* was selected for our prediction HT model to differentiate the PCOS+HT and HT groups (**Table 4**).

The specific GO categories enriched by genes related to ‘PCOS element’ are associated with female meiosis and establishment of spindle localization. In addition to the regulation of insulin secretion, oocyte meiosis was significantly associated with PCOS in a pathway-based approach and the application of meta-analysis gene-set enrichment of variant associations (MAGENTA) to the PCOS GWAS dataset (92). Mammalian oocytes undergo meiotic maturation, including extreme asymmetric division and polar body extrusion, to produce fertilizable haploid eggs. Centromere-free bipolar spindle assembly relies on the close interactions between microtubules and actin filaments, which are critical for spindle migration to the actomyosin-rich oocyte cortex and proper spindle positioning for asymmetric division (93, 94). Precise alignment and segregation of chromosomes during meiosis are crucial events that require stabilization of the spindle pole assembly by microtubule cross-linking and anchoring of the minus ends of microtubules in the spindle pole region (95). The assembly of spindles with unstable poles is a leading cause of aneuploidy, resulting in pregnancy loss and genetic defects. Mutations in meiotic genes can impair meiotic progression leading to oocyte death (96).

The PCOS prediction model included SNP rs1786263 in *CEP192* (**Table 4**), which encodes a protein essential for bipolar spindle assembly and high fidelity of chromosome segregation. Its depletion delays microtubule spindle assembly and causes chromosomal misalignment (97). Similarly, rs41298115 in *RAB6A* encoding a small GTPase was included in the PCOS model and was one of four unique variants significantly differentiating patients with combined PCOS and HT from those with PCOS alone in TaqMan genotyping (**Table 3**). Rab6A plays a key role in the maintenance of the cytoskeletal structure and normal progression of oocyte maturation (98, 99). Rab6A knockdown mouse oocytes fail to form an actin cap and microtubule network, and the spindle assembly checkpoint during meiosis is inactivated (99). Upon Rab6A depletion, spindle defects and chromosomal misalignment were significantly increased. Additionally, reduced intracellular Ca^2+^ stores and endoplasmic reticulum abundance have been observed (98). Through recruitment of the dynein/dynactin motor complex, Rab6A plays an essential role in microtubule-dependent retrograde trafficking and recycling in the Golgi apparatus. It interacts with several effector proteins localized in the Golgi called golgins (100). Interestingly, rs634710 in *GOLGA1*, which encodes golgin A1, was one of the variants selected to both final prediction models (**Table 4**).

Vesicular transport and regulation of the insulin receptor signaling pathway were represented by several GO terms associated with the ‘minimal genetic background’ variant set (**Supplemental Table S5**). Many important transmembrane receptors and transporters are selectively sorted by endosomal trafficking, in cooperation with sorting nexins and the retromer complex. The retromer protects these proteins from degradation in the lysosome, directing them back to the cell surface (recycling), Golgi network, or specialized endosomes (101). Abnormal endosomal trafficking after T cell activation leads to the loss of surface expression and, ultimately, lysosomal degradation of the TCR or glucose transporter GLUT1 (102). Variants of the syntaxin-binding protein 4 (*STXBP4*) and leucine-rich repeat kinase 2 (*LRRK2*) genes were included in our final HT model (**Table 4**); both genes are involved in vesicle trafficking. STXBP4 phosphorylation is required for insulin-stimulated Glut4 translocation and glucose uptake, suggesting that defects in STXBP4 phosphorylation underlie insulin resistance (103). With the association of the SNARE syntaxin-4, STXBP4 plays a role in the regulation of insulin release by pancreatic beta cells after stimulation by glucose (104). LRRK2 is a key regulator of Rab GTPases through phosphorylation and has been implicated in retromer-dependent protein recycling, maintenance of organelles, including Golgi, endosomes, and lysosomes, and the regulation of primary ciliogenesis. Patients with *LRRK2* mutations exhibited altered lysosomal morphology (105).

Genes assigned to variants from the ‘HT element’ were specifically enriched with GO categories related to ciliary assembly and intraciliary transport (**Supplementary Table S9**). Cilia are specialized microtubule-based organelles that, depending on their axonemal structure, are used by cells to direct fluid flow over their surface (motile cilia) or sense and conduct extracellular signals (primary cilia). Primary cilia concentrate various ion channels and receptors, including Hedgehog (Hh), Wingless (Wnt), Notch, transforming growth factor (TGF)-β, and platelet-derived growth factor receptors; hence, they play important physiological roles in embryonic development and tissue homeostasis (106). Ciliary dysfunction and disorder of their signaling activities are associated with a group of diseases known as ciliopathies, with a wide range of clinical manifestations, including, among many others, female and male infertility and cyst formation (107, 108). The primary cilia play an important role in maintaining the globular follicular structure of the thyroid gland. Their defects result in the irregular dilation of follicles and decreased colloid Tg levels (109). Thyrocyte cilia contain the Tg receptor LDL-related protein 2 (LRP2) and are involved in TSH-mediated Tg endocytosis, which is crucial for thyroid hormone release (110). LRP2 knockout mice showed hypothyroidism associated with decreased serum Tg and fT_4_ levels and increased TSH levels (111).

Ciliary and signaling components are trafficked in the anterograde and retrograde directions along the axoneme microtubule by intraflagellar transport (IFT) complexes, which engage dynein and kinesin motors, respectively (107). Recently, cyclin-dependent kinase 20 (CDK20) was shown to regulate ciliary retrograde protein trafficking by interacting with TBC1D32 and the phosphorylation of ciliogenesis-associated kinase 1 (CILK1) (112). CILK1 is involved in motor switching at the ciliary tip. In *CDK20* knockout cells, IFT proteins accumulate at the bulging ciliary tips and are eliminated as extracellular vesicles. Mutations in *CDK20* and *TBC1D32* are associated with ciliopathies and defective embryonic development resulting from the dysregulation of Hh signaling (113). In our study, the variant in *CDK20* significantly differentiated the PCOS+HT group of patients from the PCOS and HT alone groups (**Table 3**). In addition, SNP in *DYNC2H1* encoding dynein cytoplasmic 2 heavy chain 1, a component of the IFT dynein motor (114), was included in the final PCOS model (**Table 4**).

Changes in ovarian ECM composition and the organization of collagen, fibronectin, and elastin play significant roles in follicle development (115). Recently, two rare missense variants in *FBN3* encoding an ECM protein, a member of the fibrillin/latent TGF-β binding protein (LTBP) family, and a missense variant in *FN1*, which encodes a member of the fibronectin family, were identified by WES in families with PCOS (48). In addition, the *FBN3* D19S884 allele 8 variant has been identified by candidate gene analysis and its causality in PCOS susceptibility has been suggested (116, 117). Together with other components of TGF-β and androgen signaling, *FBN3* is present in the fetal ovary, and its expression is restricted to the perifollicular stroma of the follicles (118). It has been hypothesized that changes in *FBN3* expression during fetal development influence TGF-β bioactivity and collagen deposition in the ovarian ECM and predispose women to PCOS later in life (119). In turn, TGF-β is a key regulator of immune tolerance that upregulates Treg cells, and its downregulation may predispose to autoimmunity (19). Accordingly, variants in both *FBN3* (rs3829817) and *TGFB1* (rs1800472) were selected for the final HT model, and the variant rs4484951 in *FNDC7*, which encodes a fibronectin type III domain-containing protein, was selected for both prediction models in our study (**Table 4**). In addition, rs17855988 in *ELN* encoding elastin was the most significant variant (the highest RI) according to MCFS-ID analysis in the P+H *vs.* P comparison (**Supplementary Figure S1**).

SNP rs8101480 in *HIF3A* was a unique variant that significantly differentiated the PCOS+HT and HT groups (OR = 5.88; *p*_adj_ < 0.05) in both allelic and genotype analyses of the TaqMan genotyping results (**Table 3**). HIF3A belongs to the transcription factor family and is involved in adaptive responses to hypoxia. It is highly expressed in adipocytes and is negatively correlated with insulin resistance and adipose tissue dysfunction in obesity (120).

### 4.2 Prediction modeling of the joint occurrence of PCOS and HT

Based on TaqMan genotyping of WES-identified variants in a cohort of 533 patients, we created two prediction models to differentiate patients with joint PCOS and HT from those with PCOS or HT alone. Both final models included 15 genetic variants, and their prediction accuracy estimated by the AUC value was 0.78, with the specificity of 68% and 82% for the PCOS and HT models, respectively (**Tables 4**, **5**). Use of the 70% cutoff of the prediction score to distinguish patients with higher confidence improves the model parameters, increasing both the accuracy (up to 0.87) and specificity (up to 93%) of prediction, but 35-40% of the model results remain inconclusive. In addition, we identified the *FNDC7* variant as the one that contributed the most to the predictive power of both models, followed by *RAB6A* and *GOLGA1* variants in the PCOS model and *HIF3A* and *FBN3* variants in the HT model.

Our study has some limitations. One of these may be that the age of the patients with HT in our study was significantly higher than that of the patients with PCOS and PCOS+HT. Therefore, the possibility that HT appears later in patients with PCOS cannot be ruled out. On the other hand, in PCOS patients, HT may be detected earlier thanks to thyroid tests performed during the diagnostic process. Second, the sample size of our patient cohort may have been relatively small, especially considering the whole-exome analysis of sporadic cases. This may partly contribute to the poor validation of WES-derived classifiers in an independent group of patients (121). However, it must be emphasized that the WES-derived models in our study could not be fully validated owing to a partial lack of suitable probes. To increase the power of our study and the chance of detecting significant relationships, we adopted more stringent diagnostic criteria, thus reducing the phenotypic and, most likely, genotypic heterogeneity of the studied groups. Our goal was to obtain the largest possible collection of putative candidate genes to identify the biological processes involved. A study in women with sporadic diseases was supposed to facilitate the unraveling of the complex genetic background of these two polygenic disorders. Indeed, analyses of functional enrichment in the obtained gene sets indicated several processes significantly related to the etiology of PCOS and HT.

## 5 Conclusion

This study provides new insights into the joint occurrence of PCOS and HT, despite their phenotypic heterogeneity, complex genetic backgrounds, and limitations. Several variants were found to significantly differentiate between patients with joint PCOS and HT and those with both diseases separately. Functional enrichment analysis of genes related to the selected variants identified several processes whose abnormal functions may be involved in the development of both diseases, such as immune responses, insulin signaling, *O-*linked glycosylation, ECM organization and composition, membrane trafficking, ion channels, retrograde and axonemal transport, follicular development, meiosis, and cell communication, including gap junctions. Overall, our study supports the concept that PCOS and HT share a large portion of their genetic backgrounds, and the joint occurrence of both diseases may be determined by the emergence of additional variants that disrupt the same basic processes and mutually predispose patients to a second disease.

Novel candidate genes have been proposed as predisposing factors for the co-occurrence of PCOS and HT. Based on the TaqMan genotyping data for WES-selected variants, two prediction models were proposed, differentiating patients with both diseases and each disease separately, with 78% prediction accuracy. Such WES-generated models can be useful for creating multigenic panels, which may eventually be introduced into clinical practice. In the case of the joint occurrence of PCOS and HT, studies using WES can help identify patients with a predisposition to comorbidity even before the disease develop, enable the implementation of appropriate preventive measures, and support parental planning and decisions regarding childbirth.

## Data availability statement

A large portion of original data presented in the study is included as **Supplementary Material** in this manuscript. Due to the ethical restrictions and data protection regulations (possibility of identification), the raw WES data will be made available by the authors upon reasonable request.

## Ethics statement

The studies involving human participants were reviewed and approved by Local Ethics Committees at Medical University of Warsaw and Centre of Postgraduate Medical Education. The patients/participants provided their written informed consent to participate in this study.

## Author contributions

EH, KS, NZ-L and MK contributed to conception and design of the study. KS, KJ, MG and RS participated in patient recruitment. EH, KS and NZ-L organized the database. NZ-L, AK and MP performed the experiments. MK and MD performed the statistical analyses. KS, MK and MD wrote sections of the manuscript. EH acquired funds and wrote the original draft of the manuscript. EH, NZ-L, MK and KS critically revised the manuscript. All authors approved the submitted version of the manuscript.

## Supplementary material

Supplementary Figure S1 Selected variants ranked according to the relative importance (RI) value in comparisons: **(A)** PCOS+HT and PCOS groups, **(B)** PCOS+HT and HT groups.
