# Supplementary figures and images for: Exome sequencing to explore the possibility of predicting genetic susceptibility to the joint occurrence of polycystic ovary syndrome and Hashimoto’s thyroiditis
Source: Front Immunol. 2023 Jul 20;14:1193293. doi: 10.3389/fimmu.2023.1193293 (PMC10397507; doi:10.3389/fimmu.2023.1193293)

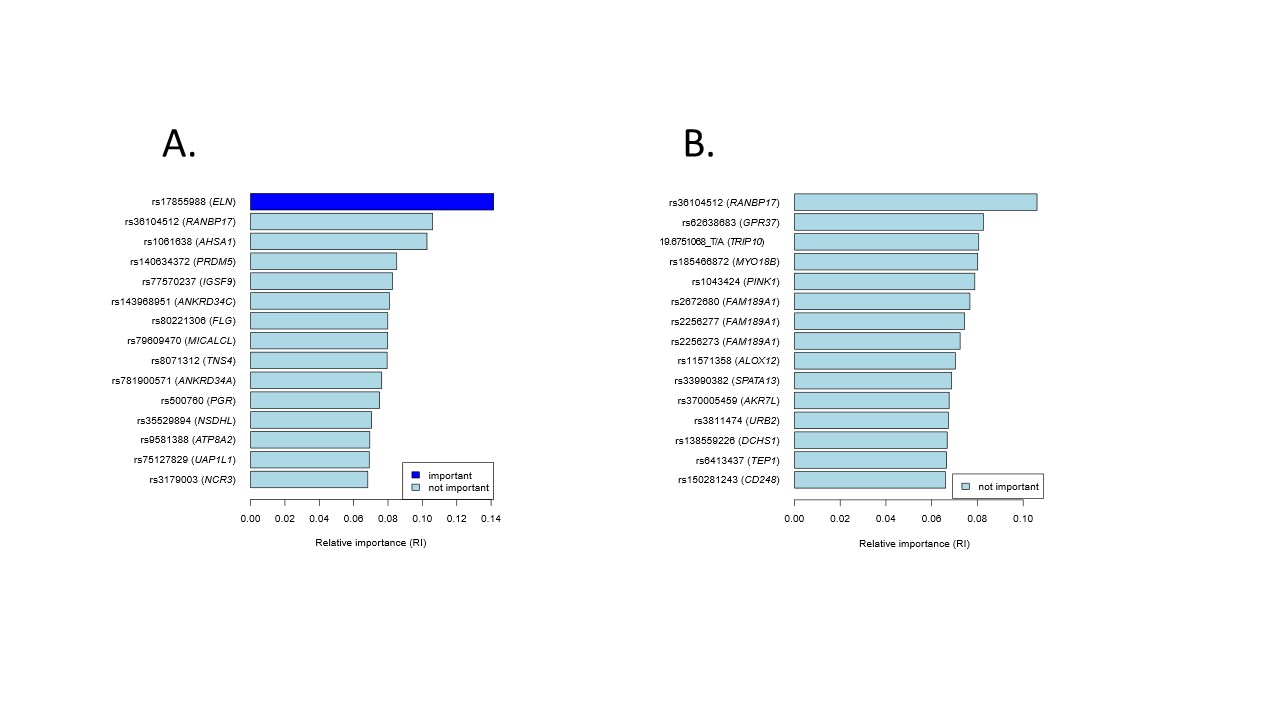

Supplement: Supplementary Figure S1 — Selected variants ranked according to the relative importance (RI) value in comparisons: (A) PCOS+HT and PCOS groups, (B) PCOS+HT and HT groups. [file Image_1.jpeg]
